# Achieving holistic care for the mentally ill: The need for more caregiver support groups in Africa

**DOI:** 10.1192/j.eurpsy.2021.1051

**Published:** 2021-08-13

**Authors:** A.J. Ogunmodede, J. Ogunmodede, O. Buhari, O. Adegunloye

**Affiliations:** 1 Dept Of Behavioural Sciences, UNIVERSITY OF ILORIN TEACHING HOSPITAL, ILORIN, Nigeria; 2 Dept Of Medicine, UNIVERSITY OF ILORIN & University OF ILORIN TEACHING HOSPITAL, ILORIN, Nigeria; 3 Dept Of Behavioural Sciences, UNIVERSITY OF ILORIN & University OF ILORIN TEACHING HOSPITAL, ILORIN, Nigeria

**Keywords:** holistic care, caregiver support group, Africa, mental healthcare

## Abstract

**Introduction:**

Caregiver support groups provide an avenue for interactions among the caregivers of the mentally ill, where they share their fears, hopes and uncertainties about their ill relatives. They are a means to be “heard” by care providers, a platform for psychoeducation as well as an avenue for participation in clinical decision making and formulation of patients’ care plans. In most parts of Africa, such support groups do not exist and where they do, they are poorly structured and poorly funded.

**Objectives:**

This review was aimed at examining the concept of caregiver support groups for the mentally ill globally as revealed in the currently avaliable body of knowledge, as well as raise awareness for the need for such groups in Africa

**Methods:**

A review of related literature was done using appropriate key words and search engines.

**Results:**

This review revealed the presence of well- structured support groups for the caregivers of the mentally ill in many parts of the world. The advantages of such groups and their contributions to the holistic care of these patients in those regions were also discussed, while suggesting a possible structure for their creation, sustainability and focus in Africa.

**Conclusions:**

The support of caregivers for the mentally ill must be given keen attention by both care providers and policy makers, with prime importance given to the creation and funding of more caregiver support groups in the continent in order to achieve quality and holisitic care for the mentally ill.

